# Malaria elimination: situation analysis of cases in India, the state of Madhya Pradesh in central India, and district Mandla of Madhya Pradesh

**DOI:** 10.3389/fpubh.2024.1363736

**Published:** 2024-04-09

**Authors:** Mrigendra P. Singh, Praveen K. Bharti, Harsh Rajvanshi, Ram S. Sahu, Himanshu Jayswar, Anup R. Anvikar, Altaf A. Lal

**Affiliations:** ^1^Malaria Elimination Demonstration Project, Mandla, Madhya Pradesh, India; ^2^Indian Council of Medical Research, National Institute of Malaria Research, New Delhi, India; ^3^Department of Health Services, Government of Madhya Pradesh, Mandla, Madhya Pradesh, India; ^4^Directorate General of Health Services, Government of Madhya Pradesh, Bhopal, Madhya Pradesh, India; ^5^Foundation for Disease Elimination and Control of India (FDEC India), Mumbai, Maharashtra, India; ^6^Sun Pharmaceutical Industries Ltd., Mumbai, India

**Keywords:** malaria elimination, MEDP, monitoring and accountability frameworks, robust surveillance, situation analysis, tribal malaria

## Abstract

India contributed approximately 66% of the malaria cases in the WHO South-East Asia region in 2022. In India, approximately 44% of cases have been reported to be disproportionately contributed by approximately 27 districts.^1^ A comparative analysis of reported malaria cases between January 2017 and December 2022 was performed in Mandla district, which is the site of a model malaria elimination demonstration project (MEDP) in Madhya Pradesh (MP), India. Compared to 2017, the decrease in malaria cases in Mandla from 2018 to 2022 was higher than MP and the rest of the country. The reduction of cases was significant in 2018, 2019, and 2021 (*p* < 0.01) (Mandla vs. MP) and was highly significant during 2018–2022 (*p* < 0.001) (Mandla vs. India). Robust surveillance and real-time data-based decisions accompanied by appropriate management, operational controls, and independent reviews, all designed for resource optimisation, were the reasons for eliminating indigenous malaria in Mandla district. The increase in infection rates during the months immediately following rains suggests that surveillance, vector control, and case management efforts should be specifically intensified for eliminating imported and indigenous cases in the near-elimination districts to work towards achieving the national elimination goal of 2030.

## Introduction

Malaria remains one of the most important public health problems globally, with an estimated 249 million cases and 608,000 malaria-attributable deaths reported in 2022. Approximately 94% of global malaria cases are contributed by African countries, while 2% of cases are contributed by countries in the World Health Organization (WHO) South-East Asia (SEA) region. India accounted for approximately 66% of the malaria cases in the WHO SEA region in 2022 (1).

During the COVID-19 pandemic between 2019 and 2020, the global burden of malaria increased by 6%, primarily due to the disruption of anti-malarial activities. In comparison, India was the only High Burden High Impact (HBHI) country that reported a 46% decrease in malaria cases between 2019 and 2020. However, there was a 50% decrease in the distribution of insecticide-treated bed nets in India in 2020 (2).

In India, approximately 44% of the reported malaria cases and 43% deaths are disproportionately contributed by approximately 27 tribal-dominated districts that comprise 5% of the country's population. Among these cases, 57.3% are identified as *Plasmodium falciparum* infections (3).

India had set the target to achieve zero indigenous malaria cases in 26 low-to-moderate endemic malaria states/Union Territories (UTs) by 2022 and eliminate malaria throughout the country by 2027. The proposed strategies included strengthening malaria surveillance, establishing the mechanism for early case detection and prompt treatment, distribution and promotion of the use of long-lasting insecticidal nets (LLIN), effective indoor residual sprays (IRS), capacity-building of community healthcare service providers, and inter-sectorial coordination. The 2022 target of zero indigenous cases was achieved by only two states/UTs of Puducherry and Lakshadweep, with Chandigarh and newly formed UT of Ladakh reporting only two cases each in 2022 (3, 4).

Inaccessible terrains, dense forest covers, perennial streams, poor socioeconomic indicators, poor health-seeking behavior, and inadequate health infrastructure are the significant challenges for malaria elimination in tribal-dominated areas of India. People living in these malaria-endemic areas have poor access to formal health facilities. Unqualified healthcare providers and traditional faith healers are often the first points of contact in the rural tribal areas, which is the primary cause of delay in prompt diagnosis and radical treatment (5).

In 2017, the MEDP was launched as a public–private partnership project between the Government of Madhya Pradesh (MP), Indian Council of Medical Research (ICMR), and Foundation for Disease Elimination and Control (FDEC) of India—a corporate social responsibility (CSR) subsidiary of Sun Pharmaceutical Industries Ltd.—to demonstrate that malaria elimination is possible in hard-to-reach, hilly, forested, and tribal-dominated areas. The MEDP's malaria operational elimination plan used the T4 (Track fever, Test fever, Treat malaria, and Track treatment) strategy, monitoring of vector control interventions, Mass Screening and Treatment (MSaT), needs-assessment followed by capacity-building, regular monitoring, and supervision for data-driven decision-making to ensure best outcomes of the resources deployed for the project (6–9).

The MEDP also estimated the burden of sub-microscopic malaria infection and the importation of cases into the district. From September 2017 to March 2021, for a total of 43 months of field operations, the MEDP achieved a 91% reduction in indigenous malaria cases with 10 consecutive months of zero transmission of indigenous malaria cases (10).

In the MEDP, the key interventions added to complement the interventions of the national programme included: (1) robust active surveillance using the T4 strategy, (2) periodic mass survey and treatment adopting the stratified clustered random sampling method, (3) molecular diagnosis of a subset of samples to estimate the burden of low-density malaria infection and asymptomatic cases, (4) supervised and quality-assured IRS and LLIN distribution for vector control efforts, (5) regular capacity-building of healthcare providers, (6) innovative information education communication/behavior change communication (IEC BCC) campaigns, and (7) robust reviews and accountability frameworks.

The present situation analysis was conducted to track malaria elimination progress in Mandla district post-MEDP and to highlight how the lessons learned could help achieve the national malaria elimination goal.

## Methods

**Figure 1:**
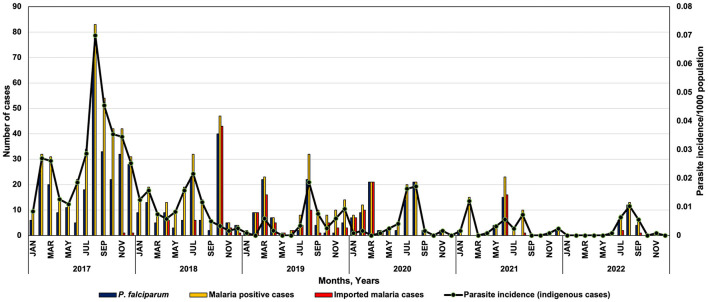
Monthly trend of *Plasmodium falciparum*, malaria cases and parasite incidence in district Mandla during 2017–2022.

This study presents the situation analysis of the reported malaria cases between January 2017 and December 2022 in Mandla district. It compares them with the reported cases in MP and India during the same period. In Mandla, the monthly malaria prevalence data were collected from the MEDP data repository, which included the active and passive cases detected using rapid diagnostic tests (RDTs), microscopic examination of blood smears, and data provided by the District Malaria Office. During the study period, the total reported malaria cases of Mandla district were classified into two groups, namely indigenous and imported cases. Annual malaria prevalence data from the state of MP and India were obtained from the official website of the National Center for Vector Borne Disease Control (NCVBDC). The annual per cent decline in malaria cases, along with a 95% confidence interval, was estimated from 2017 as a reference year. The Chi-squared test was used to compare percentage change over multiple time points between Mandla vs. MP and India. The statistical analysis was performed using R for Windows version 4.3.2. The monthly trend of malaria cases during the MEDP (January 2017–March 2021) and the post-MEDP (April 2021–December 2022) period in Mandla district is presented in Figure 1. The comparison of the annual per cent decline in malaria cases over multiple time points between Mandla district, the state of MP, and the country is shown in Table 1.

**Table 1:** Situation analysis of malaria cases during 2017 and 2022 in district Mandla, Madhya Pradesh and India.

**Year**	**Population (000)#**	**Fever cases screened**	**PF (RDT/BS)**	**PV (RDT/BS)**	**Positive (RDT/BS)**	**Imported malaria cases**	**Indigenous malaria cases**	**%decline indigenous malaria cases**	***P* value (Mandla vs. MP state and India)**
								**Base year 2017 (95% CI)**	
**Mandla**									
2017	1,186	134,025	275	134	409	2	407	Reference	
2018	1,204	108,908	125	68	193	59	134	−67.08 (−71.63, −62.28)	Reference
2019	1,211	136,005	74	52	126	60	66	−83.78 (−87.23, −79.84)	Reference
2020	1,224	64,575	82	14	96	39	57	−85.99 (−89.22, −82.24)	Reference
2021	1,241	186,502	46	16	62	17	45	−88.94 (−91.82, −85.49)	Reference
2022	1,258	213,204	23	7	30	3	27	−93.37 (−95.58, −90.49)	Reference
**MP state**									
2017	79,948	9,896,156	15,554	30,622	46,176	NA	46,176	Reference	
2018	81,090	9,817,411	6,332	15,947	22,279	NA	22,279	−51.75 (−52.21, −51.30)	< 0.0001
2019	82,232	10,069,562	3,627	10,520	14,147	NA	14,147	−69.36 (−69.78, −68.94)	< 0.0001
2020	83,374	9,056,958	3,971	2,789	6,760	NA	6,760	−85.36 (−85.68, −85.04)	0.718
2021	84,516	9,864,546	1,688	1,493	3,181	NA	3,181	−93.11 (−93.34, −92.88)	0.001
2022	85,548	11,031,117	2,452	1,374	3,826	NA	3,826	−91.71 (−91.96, −91.46)	0.228
**INDIA**									
2017	130,4457	122,422,591	533,501	308,164	841,665	NA	841,665	Reference	
2018	131,8678	124,475,724	207,198	222,730	429,928	NA	429,928	−48.92 (−49.03, −48.81)	< 0.0001
2019	1,332,900	134,230,349	156,940	181,554	338,494	NA	338,494	−59.78 (−59.89, −59.68)	< 0.0001
2020	1,347,121	97,177,024	119,088	67,444	186,532	NA	186,532	−77.84 (−77.93, −77.75)	< 0.0001
2021	1,361,343	114,391,973	101,566	60,189	161,755	NA	161,755	−80.78 (−80.87, −80.70)	< 0.0001
2022	1,373,761	152,082,808	101,070	75,453	176,523	NA	176,523	−79.03 (−79.11, −78.94)	< 0.0001

#Population of district Mandla was based on estimated population as per report of state vector borne disease programme and population of MP and India was projected population available at: https://nhm.gov.in/New_Updates_2018/Report_Population_Projection_2019.pdf. NA, Not Available; CI, Confidence Interval.

## Results

Mandla district is located at the geo-coordinates of 22° 38' 25.476” N latitude and 80° 30' 48.384 E longitude. This district is among the tribal dominant and hilly forested districts in the state of MP. Approximately 58% of the population belonged to the ethnic tribal groups, mainly “*Gond”* and “*Baiga”* (Particularly Vulnerable Tribal Group). The transmission dynamics of malaria in the district is seasonal, and *Anopheles culicifacies* is the main malaria vector that breeds in perennial streams (11).

In reference to 2017 as the base year, the per cent decrease in malaria cases in Mandla district was 67.08%, 83.78%, 85.99%, 88.94%, and 93.37% from 2018 to 2022. At the state level, in MP, the percent decrease in malaria cases during this period was 51.75%, 69.36%, 85.36%, 93.11%, and 91.71%, respectively. Similarly, during the same period in India, the per cent decrease in malaria cases was 48.92%, 59.78%, 77.84%, 80.78%, and 79.03%, respectively (Table 1).

Further analysis revealed that the per cent decrease in malaria cases in Mandla from 2018 to 2022, in reference to the year 2017, was significantly higher than MP during 2018 and 2019. The decrease was significantly higher in district Mandla than in the entire country from 2018 to 2022.

It should be noted that the data on malaria cases from district Mandla were obtained using active surveillance, passive surveillance, data from health camps, primary health centers, community health centers, district hospitals, and the sentinel surveillance network, which included registered public and private practitioners in the district. In comparison, the state and country data come solely from public (government) sources.

The monthly trend of *P. falciparum* malaria cases and parasite incidence/1,000 population showed a seasonal variation in the distribution of *Plasmodium* species in Mandla district. Most of the *P. vivax* cases were reported from March to August, showing peaks between June and August and then dominated by *P. falciparum* cases from September with peaks from October to December (Figure 1).

Mandla district reported malaria cases throughout 2017 and 2018. Zero indigenous malaria cases were reported for 3 months each in 2019, 2020, and 2021, and there were zero indigenous malaria cases for seven months in 2022. Compared to the years 2017–2020, where there were two distinct peaks of malaria in February–March and July–September, there was only one peak with much lower intensity in the July–September months of 2021 and 2022. The month-wise trend at the state and country level could not be analyzed due to the non-availability of seasonal data in the public domain.

## Discussion

The comprehensive surveillance strategy provided a robust estimate of malaria cases in Mandla district as compared to the rest of the state and the country. The MEDP also implemented MSaT to diagnose and treat the asymptomatic malaria cases during 2018–2020 and adopted the stratified clustered sampling method based on the malaria endemicity (12). Therefore, the Mandla malaria estimates were a true “total” burden of malaria as compared to the state of MP and the rest of the country. Furthermore, a higher rate of decline in malaria cases was observed during the year 2022. Most of the malaria cases were reported during the monsoon season (June–September), along with zero reported indigenous malaria cases in the consecutive 8 months of the post-intervention period of the MEDP between 2021 and 2022.

Based on the findings from the malaria elimination project in Mandla district, the significant reduction in malaria cases over 4 years is attributable to robust (active and passive) surveillance using digital tools, the T4 strategy, active monitoring of vector control interventions, periodic capacity-building of the healthcare providers, regular community mobilization, MSaT, and molecular diagnosis of a subset of samples to estimate the burden of low-density malaria infection and asymptomatic cases. The institution of appropriate management and operational controls, along with frequent internal and external reviews, contributed to prompt actions and responses based on the real-time data (10, 11, 13, 14). These protocols ensured the best outcomes of human, commodity, and financial resources used for the elimination project.

The MEDP regularly monitored and supervised vector control interventions in the district. The regular use of LLINs in the community increased from 34% (95% CI: 33.74–34.26) in 2017 to 47% (95% CI: 46.80–47.19) in 2019, and this difference was significant statistically (*p* < 0.0001). The spraying quality of the IRS improved from 47.8% in 2017 to 88.6% in 2019, and the improvement in satisfaction with the IRS by the community increased from 66.8% to 90.5% within the same period (15–17).

Alphacypermethrin was used in LLIN and IRS in Mandla. As part of the MEDP, the insecticide susceptibility tests were conducted periodically during 2017, 2018, and 2019 to regularly monitor insecticide resistance to the vector species. The results showed that alphacypermethrin was possibly resistant to the *Anopheles culicifacies* in 2017 and further developed resistance in the year 2019 (15).

The strategies used in the MEDP can serve as a guide to develop and/or refine the district-specific malaria elimination operational plans by treating the district as an operational unit as has been already proposed by NCVBDC. In addition, linking data from each district through digital systems for robust surveillance, effective supply chain management, and real-time data analysis and reporting would be highly useful for programme managers and policy makers (18).

Two additional noteworthy observations from the MEDP study that are critical to malaria elimination are: (1) the finding of 1.51% of sub-microscopic infections in the community, detected through the diagnostic PCR method, and (2) the prevalence of asymptomatic malaria that was found to be 0.98% during the mass survey conducted by the MEDP from 2018 to 2020. Based on this information, the national programme should consider testing a subset of cases using sensitive PCR methods to determine sub-microscopic infection and conduct periodic mass surveys to identify and treat asymptomatic cases during the elimination phase (19).

The incidence data for Mandla, where indigenous transmission of malaria was interrupted several times during the conduct of the project, indicates that intensified surveillance, case management, and vector control efforts immediately after rains would lead to the elimination of infections, whether they are imported or are indigenous. Additionally, the gradual increase in the malaria-free months from 2019 to 2022 indicates that the gains achieved throughout the MEDP were progressive and sustained in the district. We firmly believe that the sustained gains were attributed to robust real-time internal and external reviews as part of the programme's management and operational controls.

## Conclusion

Robust surveillance would provide information on individuals requiring treatment, including imported cases. Molecular diagnostic tests would help determine the burden of sub-microscopic/sub-RDT and asymptomatic infections. IRS and LLINs, together with minor engineering, should be deployed simultaneously, followed by an independent assessment of the households for the use of LLINs and IRS. Internal and external data quality reviews should be conducted for accountability at operational, human resource, supply chain, and scientific levels required for elimination goals.

## Data availability statement

The original contributions presented in the study are included in the article/supplementary material, further inquiries can be directed to the corresponding author.

## Author contributions

MS: Conceptualization, Data curation, Formal analysis, Writing—original draft. PB: Writing—review & editing. HR: Conceptualization, Writing—original draft, Writing—review & editing. RS: Writing—review & editing. HJ: Writing—review & editing. AA: Writing—review & editing. AL: Conceptualization, Supervision, Writing—original draft, Writing—review & editing.
